# Cannabinoid receptor type-2 stimulation, blockade, and deletion alter the vascular inflammatory responses to traumatic brain injury

**DOI:** 10.1186/s12974-014-0191-6

**Published:** 2014-11-22

**Authors:** Peter S Amenta, Jack I Jallo, Ronald F Tuma, D. Craig Hooper, Melanie B Elliott

**Affiliations:** Department of Neurological Surgery, Thomas Jefferson University Hospital, 1020 Locust Street, Thomas Jefferson University, Philadelphia, PA 19107 USA; Department of Cancer Biology, Thomas Jefferson University Hospital, 1020 Locust Street, Thomas Jefferson University, Philadelphia, PA 19107 USA; Department of Physiology, Temple University School of Medicine, 3500 N Broad St, Philadelphia, PA 19140 USA

**Keywords:** Controlled cortical impact, Traumatic brain injury, Cannabinoid, Inflammation, Intracellular adhesion molecule

## Abstract

**Background:**

Immunomodulatory therapies have been identified as interventions for secondary injury after traumatic brain injury (TBI). The cannabinoid receptor type-2 (CB_2_R) is proposed to play an important, endogenous role in regulating inflammation. The effects of CB_2_R stimulation, blockade, and deletion on the neurovascular inflammatory responses to TBI were assessed.

**Methods:**

Wild-type C57BL/6 or CB_2_R knockout mice were randomly assigned to controlled cortical impact (CCI) injury or to craniotomy control groups. The effects of treatment with synthetic, selective CB_2_R agonists (0-1966 and JWH-133), a selective CB_2_R antagonist, or vehicle solution administered to CCI groups were assessed at 1-day after injury. Changes in TNF-α, intracellular adhesion molecule (ICAM-1), inducible nitric oxide synthase (iNOS), macrophage/microglial ionized calcium-binding adaptor molecule, and blood-brain-barrier (BBB) permeability were assessed using ELISA, quantitative RT-PCR, immunohistochemistry, and fluorometric analysis of sodium fluorescein uptake. CB_2_R knockouts and wild-type mice with CCI injury were treated with a CB_2_R agonist or vehicle treatment.

**Results:**

TNF-α mRNA increased at 6 hours and 1 to 3 days after CCI; a CB_2_R antagonist and genetic knockout of the CB_2_R exacerbated TNF-α mRNA expression. Treatment with a CB_2_R agonist attenuated TNF-α protein levels indicating post-transcriptional mechanisms. Intracellular adhesion molecule (ICAM-1) mRNA was increased at 6 hours, and at 1 to 2 days after CCI, reduced in mice treated with a CB_2_R agonist, and increased in CB_2_R knockout mice with CCI. Sodium fluorescein uptake was increased in CB_2_R knockouts after CCI, with and without a CB_2_R agonist. iNOS mRNA expression peaked early (6 hours) and remained increased from 1 to 3 days after injury. Treatment with a CB_2_R agonist attenuated increases in iNOS mRNA expression, while genetic deletion of the CB_2_R resulted in substantial increases in iNOS expression. Double label immunohistochemistry confirmed that iNOS was expressed by macrophage/microglia in the injured cortex.

**Conclusion:**

Findings demonstrate that the endogenous cannabinoid system and CB_2_R play an important role in regulating inflammation and neurovascular responses in the traumatically injured brain. CB_2_R stimulation with two agonists (0-1966 and JWH-133) dampened post-traumatic inflammation, while blockade or deletion of the CB_2_R worsened inflammation. Findings support previous evidence that modulating the CB_2_R alters infiltrating macrophages and activated resident microglia. Further investigation into the role of the CB_2_R on specific immune cell populations in the injured brain is warranted.

## Introduction

Traumatic brain injury (TBI) affects over 1.4 million Americans annually, with many suffering fatal or permanently disabling injuries [1,2]. Blood-brain-barrier (BBB) disruption, a result of the post-traumatic inflammatory response, is a proposed mechanism of secondary injury and contributes to cell death or dysfunction, worsening neurologic function, and ultimately, to poorer clinical outcome [3,4]. It is also well-recognized, however, that this same inflammatory response plays an important role in the processes necessary for repair and recovery [5]. The initial traumatic insult induces the release of pro-inflammatory cytokines and chemokines triggering endothelial cell activation, chemoattractant signaling, and immune cell infiltration [6]. The release of TNF-α up-regulates the expression of intracellular adhesion molecule-1 (ICAM-1), which promotes the adherence of immune cells to the endothelium and subsequent transmigration to sites of inflammation [7]. The result of an inflammatory-driven barrier breakdown is an enhancement of a cytotoxic environment in the setting of already compromised neurons [8-10]. Infiltrating immune cells and resident microglia have been shown to demonstrate opposing pro- and anti-inflammatory phenotypes [11,12]. Pro-inflammatory cell phenotypes release cytokines and express enzymes such as inducible nitric oxide synthase (iNOS) that generate damaging free radicals and further disrupt BBB function. Anti-inflammatory phenotypes produce cytokines and growth factors that down-regulate free radical generating pathways, which can promote healing and regeneration. Optimal modulation of the post-traumatic inflammatory response will limit damage and promote reparative interactions between the immune and nervous systems.

A number of cellular targets have been identified as potential therapeutic interventions for the post-traumatic inflammatory response. The endocannabinoid system, as documented previously, represents a specialized group of endogenous neurotransmitters with a broad range of function [13]. In particular, the cannabinoid receptor type-2 (CB_2_R), expressed predominantly by circulating immune cells and resident microglia, plays an important role in the immune response to injury. Upon stimulation with its ligand, the CB_2_R possesses potent immunomodulatory and anti-inflammatory properties as reviewed by Cabral *et al*. [14-18]. In a series of studies, stimulation of the CB_2_R has been shown to dampen post-TBI inflammation including infiltrating/resident immune cell activation and neurovascular disruption, all of which were accompanied by improved functional outcome following TBI and spinal cord injuries [19-23]. These findings are supported by other laboratories, showing treatment with a CB_2_R agonist results in immunomodulation and neuroprotection in models of brain injury and neurodegeneration [18,24].

Acute immunomodulation through CB_2_R ligands is associated with improvements in outcome in animal models of brain and spinal cord injury, Huntington’s disease, and Parkinson’s disease [18-20,22-25]. Emerging evidence points to the modulation of microglia and infiltrating macrophages as a key component in CB_2_R-mediated improvements in functional outcome. Furthermore, these same immunomodulatory processes have also been implicated as important moderators underlying the histopathologic changes observed in cases of improved outcome following injury [18,19,22-24,26,27]. Microglial activation and inflammation in the traumatized brain can persist for years after TBI [28,29], and like macrophages, express pro-inflammatory (M1) and anti-inflammatory (M2) phenotypes [11,12,30]. A ‘loss of function’ in the M2 cell phenotype may underlie chronic inflammation such as occurs with traumatic brain injury [11]. Promoting the healing function of M2 immune cells through CB_2_ receptor stimulation may inhibit the detrimental effects of long-term inflammation including synapse loss, neuronal degeneration, and cognitive function [11]. The present study further investigates the role of the CB_2_R in regulating acute inflammatory vascular responses to TBI using CB_2_ receptor stimulation, blockade and deletion. We also report the evidence linking CB_2_R stimulation and alteration of the inflammatory cell phenotype.

## Methods

### Animal care and housing

Prior to initiating any research, the Thomas Jefferson University Institutional Animal Care and Use Committee (IACUC) reviewed and approved the research protocol and the use of male C57BL/6 mice. Animal care and use was monitored by the University Animal Care and Use Committee to assure compliance with the provisions of Federal Regulations and the NIH ‘Guide for the Care and Use of Laboratory Animals’. All mice were housed in the Thomas Jefferson University Laboratory Animal Services Facility which is accredited by the American Association for the Accreditation of Laboratory Animal Care and complies with NIH standards.

### Experimental design

**Figure 1:**
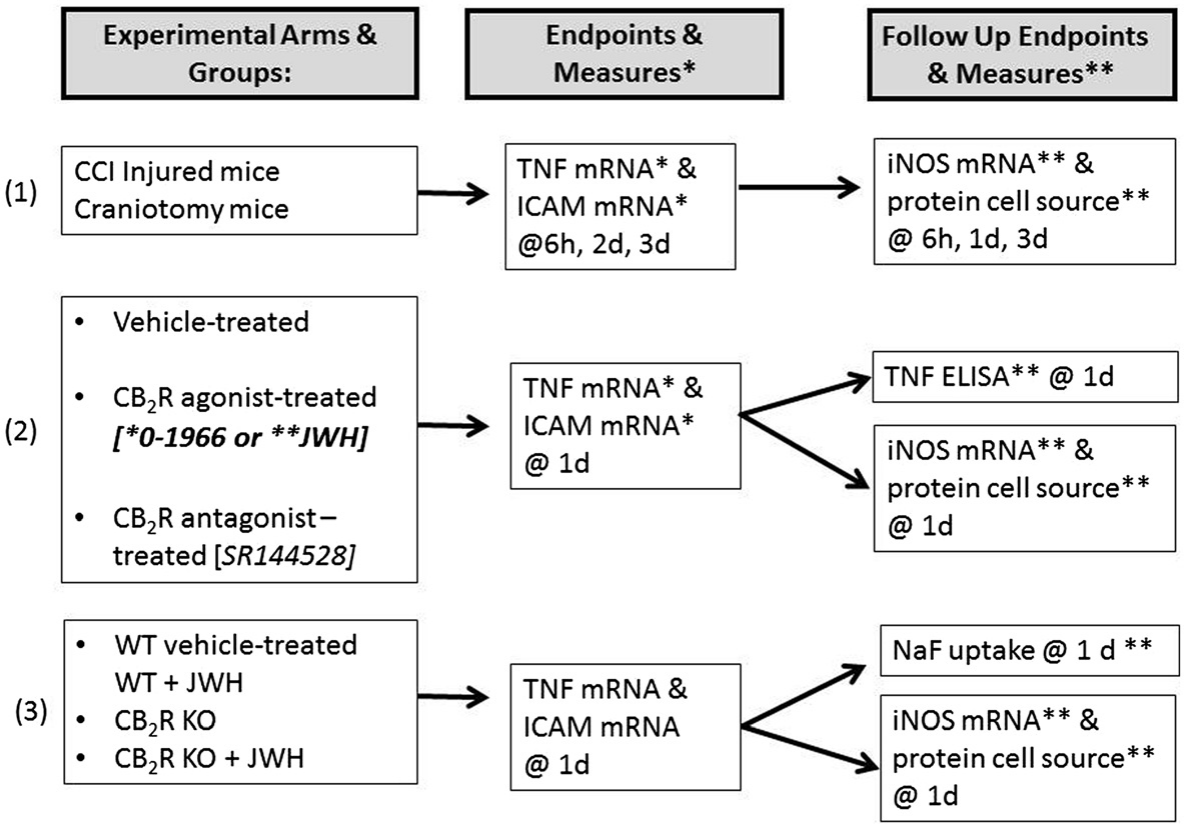
**Experimental design flowchart showing experimental groups, endpoints, and outcome measures under each experimental arm: (1) CCI injured groups over time compared to craniotomy (control), (2) CCI injured mice treated with vehicle, cannabinoid receptor type 2 (CB**_**2**_ R) agonist, (*0-1966 or **JWH-133 (JWH)), or CB_**2**_ **R antagonist (SR144528), and (3) wild-type CCI mice treated with vehicle or JWH, compared to CB**_**2**_ **R knockout CCI injured mice with and without JWH.**

Seventy-four (n = 74) adult male mice at approximately 8 weeks of age (weighing 22 to 24 g), including strains of C57BL/6 wild-type mice (Charles River, Wilmington, MA, USA) or CB_2_R knockout B6.129P2-*Cnr2*^*tm1Dgen*^/J mice (Taconic, Hudson, NY, USA) were randomly assigned to undergo controlled cortical impact injury (n = 66) or serve as craniotomy controls (control) (n = 8). There were three study arms to determine the effects of CB_2_R modulation on genes and proteins expression for primary vascular inflammatory markers (TNF-α, ICAM, iNOS, and BBB permeability) which included: (1) CCI injury over time compared to craniotomy, (2) CB_2_R agonists and CB_2_R antagonist compared to vehicle-treated mice, and (3) CB_2_R knockout (CB_2_R KO) CCI groups with and without JWH-133 compared to wild-type CCI (Figure 1). Endpoints for CCI time course experiments were at either 6 hours (n = 4) or 1 (n = 10), 2 (n = 7), or 3 (n = 5) days after CCI injury. Two administrations of synthetic selective CB_2_R agonists, 0-1966 (n = 8) or JWH-133 (n = 12), a selective CB_2_R antagonist, SR144528 (n = 4), or vehicle solution (n = 10) were administered to wild-type CCI mice as described below. To determine the selectivity for the CB_2_R, knockout mice lacking the CB_2_R were treated with a CB_2_R agonist JWH-133 (n = 3) and 0-1966 (n = 4) or vehicle (n = 9) and compared to wild-type CCI mice (n = 8). Controls underwent all surgical procedures including an equal time of isoflurane exposure, buprenorphine injection, incision and craniotomy but were not subjected to CCI injury, did not receive treatment or vehicle, and were euthanized at 3 days post-operatively. All surgeries and experimental *post-mortem* procedures were performed so that, within each cohort of mice, craniotomy or vehicle-treated control groups were run in parallel with their respective experimental groups to insure consistent environmental conditions. On an annual basis, there is a 6 to 8% mortality rate for our CCI injury model due to the formation of fatal hematomas or cerebrovascular blood clots. Controlled cortical impact injury (CCI) injury resulted in a loss of 6 of the original 66experimental CCI mice (9% mortality rate) equally distributed among groups, and final group sizes were reported for each experimental outcome (see figures).

### Traumatic brain injury

Traumatic brain injury was induced using CCI injury, a highly reproducible non-penetrating brain injury model [31]. Mice were injured using methods previously described by our laboratory [19,23,32]. Anesthesia was induced with 3% isoflurane and maintained throughout the procedure at a dose of 2.5% isoflurane. Prior to the start of the procedure, all mice were injected with short-acting buprenorphine (0.01 cc subcutaneous) for acute post-operative pain control. A right-sided 4 mm craniotomy was performed at 1 mm posterior to bregma exposing the mouse somatosensory cortex. A rounded aluminum 3 mm diameter stereotaxic impactor tip (MyNeuroLab, St. Louis, MO, USA) was used to produce a cortical injury at a 1.0 mm depth, 3 m/s velocity, and 100 msec contact time. Following injury, the bone flap was sealed with permanent cyanoacrylate-based fast-acting adhesive closures and the skin was closed with 6.0 silk sutures. Post-operative care included warming with indirect heat from a heat lamp until ambulation resumed, and unlimited access to food and water. Brain and core body temperature were maintained at 37 ± 0.5°C throughout the procedure and monitored with temporalis muscle and rectal temperature probes to avoid the neuroprotective effects of anesthesia-induced hypothermia.

### Treatment administration

Stimulation of the CB_2_ receptor was performed using agonists 0-1966 (Organix Inc., Woburn, MA, USA) or JWH-133 (Tocris Bioscience, Minneapolis, MN, USA). 0-1966 was used for the TNF and ICAM PCR experiments, while JWH-133 was used in all other experiments (Figure 1). The CB_2_R agonist was switched to JWH-133 in the later experiments in this study because it had the same selectivity profile for CB_2_R as 0-1966 but with better solubility, was easier to administer, and was commercially available as a solution. 0-1966 was dissolved in a pure ethanol:emulphor:normal saline solution (1:1:18) resulting in a final concentration of 0.5 mg/mL. The CB_2_R agonist, 0-1966, also known as 0-1966A, is an analog of bicyclic resorcinols (dimethoxy-resorcinol-dimethylheptyl) and structurally similar to cannabidiol as described by Wiley *et al*. [33]. 0-1966 demonstrates 225-fold higher selectivity for the CB_2_R (*K*i = 23 ± 2.1 nM) compared to CB_1_R (*K*i = 5,055 ± 984 nM) [33]. JWH-133 selective CB_2_R agonist (Ki = 3.4 nM), in water-soluble emulsion Tocrisolve TM 100 (Tocris Catalog Number 1684) has appoximately 200-fold selectivity over CB1 receptors. The times for repeated intraperitoneal (ip) injections of 0-1966 (5 mg/kg) and JWH-133 (1 mg/kg) for the one-day endpoint were at either 2 or 18 hours post-CCI. The timing of treatment administrations were based on our previous studies [19]. Dosages were based both on preliminary dosing studies performed by our laboratory for our TBI model as well as on previous studies on models of stroke and spinal cord injury [19-22,34]. Vehicle solution was prepared in an identical manner to include 0.2 mL of pure ethanol:emulphor:normal saline solution (1:1:18) minus the cannabinoid and administered at the same time points as 0-1966. The selective CB_2_R antagonist, SR144528, (Cayman Chemical, Ann Arbor, MI, USA) was dissolved in DMSO:emulphor:normal saline solution (1:1:18) and injected at 5 mg/kg at 2 and 18 hours post-CCI.

### BBB assessment

Fluid-phase BBB permeability was assessed using sodium fluorescein (NaF) at 1 day post-CCI in wild-type, CB_2_R KO mice with and without a CB_2_R agonist (JWH-133) or controls as previously described by our laboratories [8]. NaF uptake assay was performed for 20 mice randomly divided into CCI subgroups euthanized at either 1 day (n = 236) or serving as controls (n = 4). CCI subgroups consisted of wild-type treated with vehicle or JWH-133, and CB_2_R KO receiving vehicle or JWH-133. Brain samples were run in duplicate experiments. We selected NaF to evaluate changes in BBB permeability due to its low molecular weight (376 Da) compared to others probes that bind to albumin such as Evans Blue, horseradish peroxidase (HRP), or IgG (≥67,000 Da). Thus, NaF is a more sensitive probe that allows for detection of smaller leaks in the barrier. Mice were injected ip with 100 μL of 10% NaF in PBS and the NaF was allowed to circulate for 10 minutes. Following administration of a lethal dose of sodium pentobarbital, cardiac blood was collected followed by transcardial perfusion with 15 mL of heparin (1,000 U/L) in PBS. Brains were sectioned into a left and right hemisphere and micro-dissected to separate the cerebral cortex, and processed immediately. To determine BBB permeability, tissues were weighed, homogenized in 1/10 dilution in PBS and centrifuged at 14,000 × g for 2 minutes. Five-hundred microliters of the clarified supernatant was transferred into 500 μL of 15% trichloroacetic acid and centrifuged at 10,000 × g for 10 minutes while the pellet was retained for RNA isolation. One hundred and twenty-five microliters of 5 N NaOH was added to 500 μL of the supernatant, and the amount of fluorescein for each sample was determined using standards ranging from 125 to 4,000 μg on a Cytofluor II fluorometer (PerSeptive Biosystems, Framingham, MA, USA). Serum levels of sodium fluorescein were assessed as previously described so that signals in CNS tissue samples could be normalized against the amount present in the circulation. NaF uptake into each brain region of interest is expressed as (ug/g NaF in cortex)/(μg NaF in serum).

### RT-PCR

The pellet isolated during the BBB assessment outlined above was subsequently used for RNA isolation. Total RNA was extracted with the RNeasy Miniprep Kit (Qiagen, Valencia, CA, USA), reverse transcribed into cDNA with MMLV reverse transcriptase (Promega, Madison, WI, USA) and then measured by quantitative real-time PCR with gene-specific primers and probes [35]. IQ supermix and the iCycler iQ real-time detection system were also used for quantification (Bio-Rad Laboratories, Hercules, CA, USA). All samples were run in duplicate and compared to cDNA gene standards to determine copy numbers, which were normalized to the copy number of each sample’s housekeeping gene L13. Levels of mRNA are reported as the fold change in gene expression of normalized to the endogenous reference gene L13 and relative to the untreated, craniotomy controls.

### Immunohistochemistry

Mice were administered a lethal dose of sodium pentobarbital (120 mg/kg, ip) and underwent cardiac perfusion with heparinized saline followed by 4% paraformaldehyde. Brains were post-fixed in 4% paraformaldehyde for 24 hours, then transferred to 30% sucrose for storage. Frozen sections were cut coronally with a cryostat at –24°C (20 μm thick), and air dried overnight. Tissues were incubated in 10% NGS in O.3% Triton-100. Coronal brain sections were labeled using the following primary antibodies overnight at room temperature: (1) rabbit anti-mouse iNOS (1:200; Enzo Life Science, Farmingdale, NY, USA) and (2) rabbit anti-mouse ionized calcium-binding adaptor molecule-1 (Iba-1) (1:250; Wako Pure Chemical Industries, Richmond, VA, USA). Fluorescent secondary antibodies DyLight 488- or 549-conjugated AffiniPure Goat anti-rabbit IgG (Jackson ImmunoResearch, West Grove, PA, USA) were applied for 2 hours at room temperature. Negative control staining was performed by omitting the primary antibodies.

### Statistical analyses

All statistical analyses were performed using the GraphPad Prism 5 software program (La Jolla, CA, USA). To determine differences between CCI injury and controls at 6 hour, 2 day and 3 day time points, differences between wild-type and knockout mice, and and differences between vehicle-treated, agonist-treated, and antagonist-treated groups, statistical comparisons were performed using a one-way ANOVA followed by Bonferroni *post hoc* analysis for experimental groups compared to control. Significance levels were set at *P <* 0.05 for all statistical analyses and results are reported as the mean and SEM.

## Results

### TNF-α

**Figure 2:**
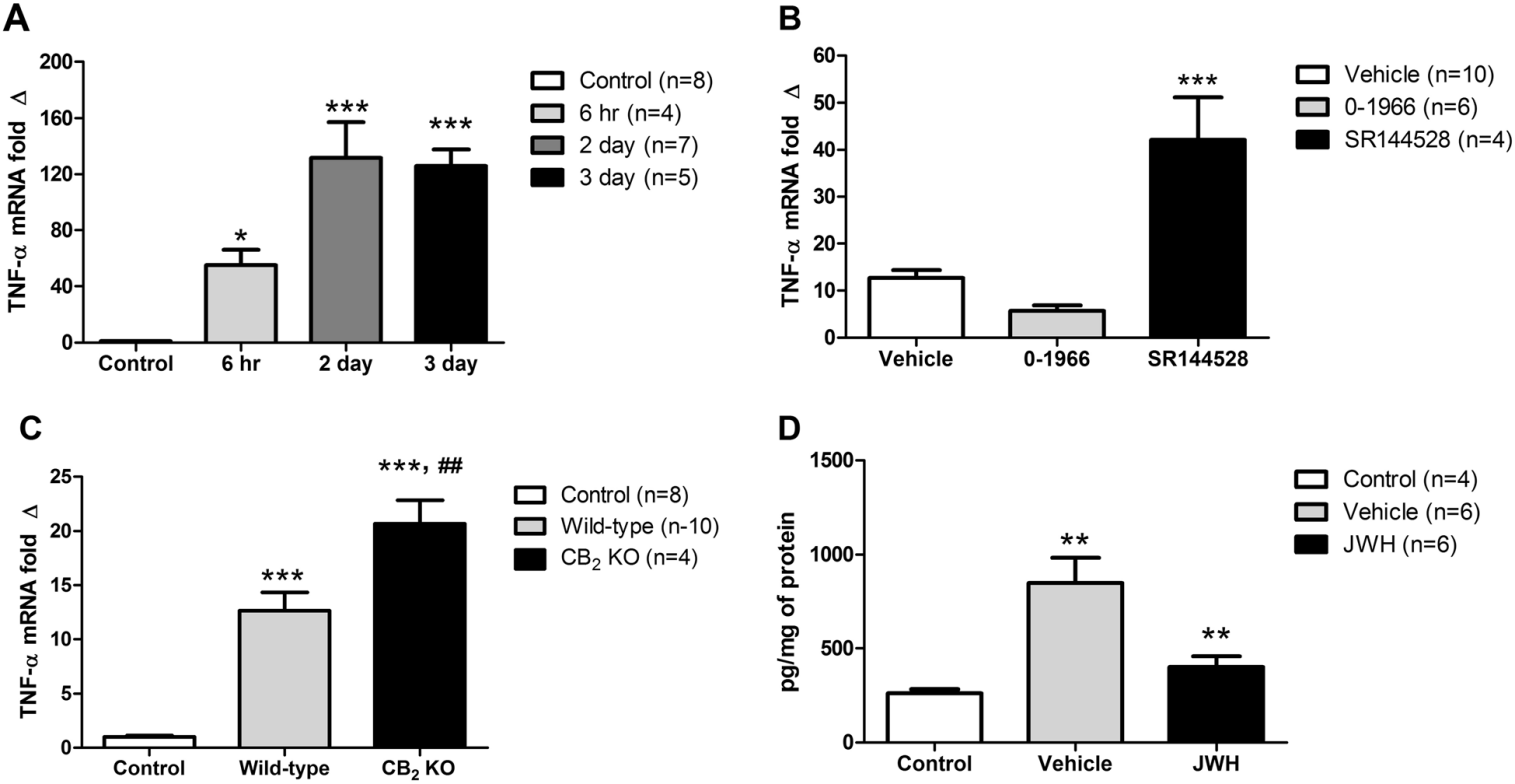
**TNF-α mRNA in the injured cortex measured using quantitative real-time PCR (A-C) and TNF-α protein concentration using an enzyme-linked immunosorbent assay (D). (A)** TNF-α mRNA at 6 hours, 2 and 3 days after CCI injury compared to craniotomy (control), *P < 0.05 and ***P < 0.001. **(B)** TNF-α mRNA at 1 day after CCI injury in wild-type mice treated with a vehicle solution (vehicle), cannabinoid receptor type-2 (CB_2_) agonist (0-1966) or CB_2_ receptor antagonist (SR144528) compared to vehicle control, ***P < 0.001. **(C)** TNF-α mRNA at 1 day after CCI injury in CB_2_ knockout (CB_2_ KO) and wild-type mice compared to control, ***P < 0.001, and ##*P* < 0.01 compared to wild-type mice. **(D)** TNF-α protein concentration at 1 day post-CCI in vehicle-treated and CB_2_ agonist-treated (JWH-133) mice compared to control and vehicle, respectively, **P < 0.01.

The expression of mRNA specific for the pro-inflammatory cytokine TNF-α was significantly increased by comparison with controls at each time points examined after CCI in wild-type mice including 6 hours (*P* < 0.05), 1 day (*P* < 0.001), 2 and 3 days (*P* < 0.001) (Figure 2A and 2C). Treatment with a CB_2_R antagonist, SR144528, significantly increased TNF-α mRNA expression at 1 day post-CCI (ANOVA *P* < 0.0001, F = 23.00), but mRNA levels where not altered after administration of a CB_2_R agonist, 0-1966 (Figure 2B). The increase in TNF-α mRNA mediated by the antagonist are paralleled by genetic deletion of the CB_2_R at one day post-CCI compared to control (*P* < 0.001) and wild-type mice, *P* < 0.01 (Figure 2C). As shown in Figure 2D, the elevation in the levels of TNF-α protein at 1 day following CCI were reduced by CB_2_R agonist treatment (Figure 2D).

### ICAM

**Figure 3:**
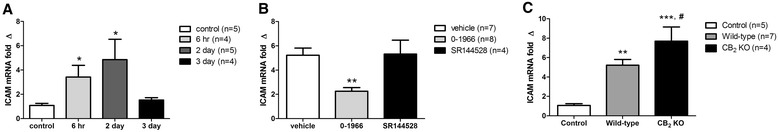
**Intracellular adhesion molecule (ICAM) mRNA expression in the injured cortex measured using quantitative real-time PCR. (A)** ICAM mRNA at 6 hours, 2 and 3 days after CCI injury compared to control, *P < 0.05. **(B)** ICAM mRNA at 1 day after CCI injury in wild-type mice treated with a cannabinoid type-2 (CB_2_) agonist (0-1966) and CB_2_ antagonist (SR144528) compared to vehicle-treated CCI controls, **P < 0.01. **(C)** ICAM mRNA at 1 day after CCI injury in CB_2_ knockout (CB_2_ −/−) mice and wild-type mice compared to control, **P < 0.01 and ***P < 0.001; ICAM mRNA post-CCI in CB_2_ knockout (CB_2_ −/−) mice compared to wild-type mice, #*P* < 0.05.

Increases in intracellular adhesion molecule-1 (ICAM-1) induced by increases in TNF-α were expected to occur in the first few days after injury when macrophage and microglial infiltration to the injured site peaks. ICAM mRNA was found to be significantly increased in the injured cortex at 6 hours, 1 and 2 days compared to controls ANOVA *P* < 0.05 (Figure 3A and 3C). ICAM mRNA expression returned to control levels by 3 days after injury. Administration of a 0-1966 significantly reduced ICAM-1 mRNA at one day post-CCI (ANOVA *P* < 0.01 and F = 9.53), while treatment with a CB_2_R antagonist, SR144528, did not change mRNA levels (Figure 3B). CCI induction in genetically modified mice lacking the CB_2_R KO significantly increased ICAM-1 mRNA expression at 1 day post-CCI compared to wild-type mice with and without CCI (ANOVA *P* < 0.001 F = 16.63) (Figure 3C).

### BBB permeability

**Figure 4:**
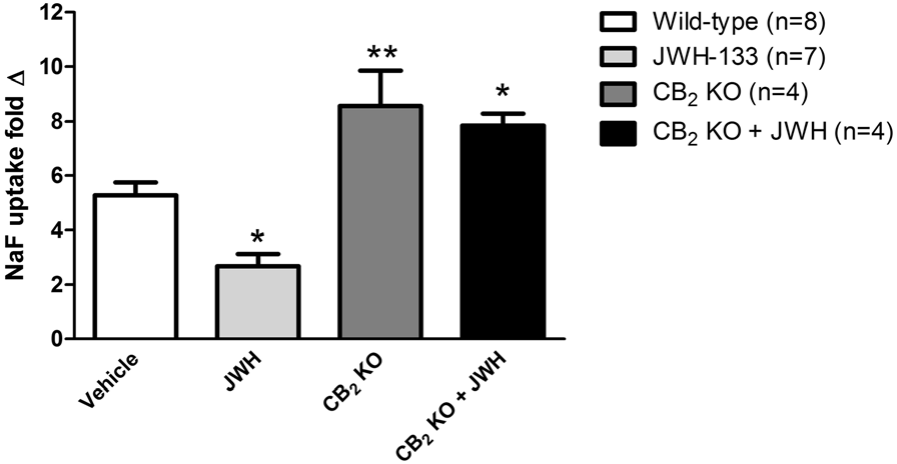
**Blood-brain-barrier permeability assessment using sodium fluorescein (NaF) uptake in the injured cortex.** NaF uptake for wild-type CCI injured mice treated with vehicle compared to JWH-133 (JWH), *P < 0.05 and cannabinoid type-2 knockout CCI mice (CB_2_R KO) treated with vehicle or JWH, **P < 0.01 compared to wild-type vehicle-treated CCI mice (vehicle).

BBB permeability was assessed by sodium fluorescein (NaF) uptake into the injured cortex (Figure 4). Treatment with JWH-133 reduced the injury-induced increase in NaF uptake, *P* < 0.05. NaF uptake was significantly increased in CCI injured CB_2_R KO mice treated with the CB_2_R agonist or vehicle (*P* < 0.05; *P* < 0.01) when compared to CCI-injured wild-type mice. The receptor selectivity of the agonist at the BBB was demonstrated as CB_2_R KO mice treated with CB_2_R agonist, JWH-133, were not different from CB_2_R KO mice treated with vehicle control.

### iNOS

**Figure 5:**
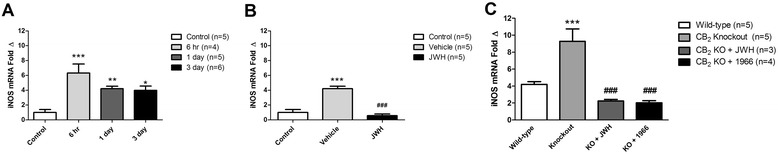
**Inducible nitric oxide synthase (iNOS) mRNA in the injured cortex. (A)** iNOS mRNA at 6 hours, 1 and 3 days after CCI injury compared to control, *P < 0.05, **P < 0.01, and ***P < 0.001. **(B)** iNOS mRNA at 1 day after CCI injury in mice compared to control, *P* < 0.001, and CCI mice treated with JWH-133 (JWH) compared to vehicle, ###*P* < 0.001. **(C)** iNOS mRNA in CB_2_ knockout (CB_2_ KO) mice compared to wild-type mice at 1 day after CCI injury, ***P < 0.001, and CB_2_ KO treated with JWH and 0-1966 (1966) compared to CB_2_ KO mice ###*P* < 0.001.

iNOS mRNA was significantly increased in the injured cortex at 6 hours, 1 day, 2, and 3 days after injury (*P* < 0.001; F = 10.57) compared to controls (Figure 5A). Increased iNOS mRNA expression in vehicle-treated mice at 1 day post-CCI was attenuated by treatment with a CB_2_R agonist (JWH-133) ANOVA *P* < 0.001 and F = 38.35 (Figure 5B). Interestingly, CCI injury in knockout mice lacking the CB_2_R resulted in a considerable increase in iNOS mRNA levels (nearly 10-fold increase) in the injured cortex than in CCI injured wild-type mice (ANOVA *P* < 0.0001 and F = 20.67). Treatment with agonists JWH-133 and 0-1966 reduced the levels of iNOS in CB_2_R KO mice indicating mechanisms other than the CB_2_R are involved in the agonist’s actions in the injured parenchyma.

**Figure 6:**
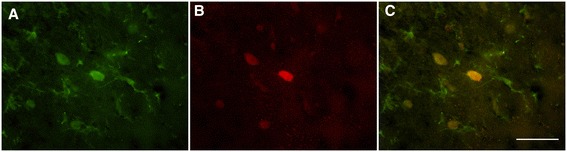
**Macrophage/microglial marker, Iba-1, and inducible nitric oxide synthase (iNOS) immunofluorescence in the remaining cortical area adjacent to the contusion.** Image shows **(A)** Iba-1 positive cells (green), **(B)** iNOS positive cells (red), and **(C)** merged image showing Iba-1 co-localized with iNOS positive cells with a retracted, amoeboid morphology (yellow).

Immunohistochemical double labeling for iNOS and a macrophage/microglial specific marker, ionized calcium-binding adaptor molecule (IBA-1) was used to qualitatively evaluate the cellular source of iNOS in the injured cortex (Figure 6). Cells within the cortical tissue surrounding the contusion that were positive for iNOS were co-labeled as macrophage/microglia cells. Some iNOS positive cells were also found distal to the injury in the cingulate cortex and subcortical areas; however, these cells did not co-localize with IBA-1 and showed a neuronal morphology (not shown).

## Discussion

Previously, our laboratory found CB_2_R stimulation reduced BBB permeability and decreased the number of macrophage/microglia in mice with controlled cortical injury [19,23]. We now show that early treatment with a CB_2_R agonist reduces the post-traumatic increase in intracellular adhesion molecule (ICAM-1) mRNA expression after TBI. This effect is accompanied by reduced levels of TNF-α protein. Conversely, genetic deletion of the CB_2_R increased the expression of ICAM-1 and TNF-α mRNA and exacerbated the BBB permeability that follows TBI. Pharmacological blockade of the CB_2_R with an antagonist also increased the levels of TNF-α mRNA. CB_2_R stimulation improved BBB integrity after TBI, likely secondary to an attenuation of endothelial cell, macrophage, and microglia activation [19,23]. The failure of the CB_2_R agonist to impact these barrier responses in mice lacking the CB_2_R demonstrates that the mechanism of action, at least peripherally or at the blood-brain interface, is selective for the CB_2_R. Interesting and not entirely surprising findings were that both CB_2_R agonists in CB_2_R KO mice showed similar effects in reducing iNOS mRNA after injury. This finding indicates the involvement of a non-CB_2_ receptor, although due to the low affinity for the CB_1_ receptor, activation of the CB_1_ receptor is unlikely to be involved but cannot be completely ruled out. JWH-133 improved cerebral infarction but the effect was absent in CB_2_R KO mice. [24]. Although there is some overlap between secondary injury mechanisms for stroke and traumatic brain injury, they are in fact distinguishable. CB_2_R selective effects on cell infiltration and BBB permeability make a different contribution to the damaged brain in stroke compared to TBI. We conclude that the effects of the drug appear to have CB_2_R selectivity peripherally and at the BBB interface; however, once penetrating the brain, there may be non-CB_1_ and non- CB_2_ receptors involved. Synthetic CB1/CB2 receptor ligands can interact with non-CB_1_R, non-CB_2_ G protein-coupled receptors, transmitter gated channels, ion channels and/or nuclear receptors and is reviewed by Pertwee *et al*. [36]. While the G protein-coupled receptor GPR55 and potassium channels have been excluded from the actions of JWH-133, others have not yet been elucidated. These findings provide additional support for the concept that the endogenous cannabinoid system facilitates protection against secondary injury following TBI [37,38], and that the neuroprotective effects of CB_2_R stimulation are due, in part, to the modulation of intracranial microvascular function leading to attenuated immune cell infiltration. Similar effects have been reported in studies of ischemic brain injury, whereby reductions in immune cell trafficking accompanied CB_2_R agonist-induced decreases in ICAM-1 expression and BBB damage [20,21,34]. Moreover, following exposure to a variety of inflammatory stimuli, CB_2_R stimulation increased endothelial cell tight junction protein expression in the brain microvasculature and reduced vascular permeability and the expression of ICAM-1 and VCAM-1 [39]. Finally, genetic knockout of the CB_2_R has been shown to worsen inflammation, injury and behavioral deficits in several models of CNS diseases [18,20,25,39]. These findings indicate the importance of the CB_2_R in regulating the immune and vascular response, as well as recovery following injury to the brain.

Early, rapid and robust production of pro-inflammatory cytokines, such as TNF-α after TBI, is responsible for the upregulation of ICAM-1 expression and subsequent immune cell infiltration to the injured brain [7,40]. TNF-α is a key cytokine that has been implicated in the induction of pathological inflammation in a variety of models including TBI. The finding that TNF-α expression following CCI is strongly elevated by administration of a CB_2_ antagonist together with its increase in CB_2_ knockout mice subject to CCI, confirms the importance of the CB_2_ pathway in controlling TNF-α expression after TBI. The lack of changes in TNF message transcription with CB_2_R stimulation in this study may be secondary to the post-CCI time point examined. Treatment with a CB_2_R agonist reduced TNF-α mRNA at 15 hours after middle cerebral artery occlusion [24]. Fluctuations in TNF-α protein levels have been reported hours to days after CCI injury [41-43]. Cyclical changes in cytokine genes including TNF-α have also been shown after CCI injury in mice [6]. TNF-α mRNA fluctuates over time after injury, in which the levels, albeit increased compared to controls, were at the lowest level at the 1 day time point.

It is important to note that the TNF-α protein levels were reduced by CB_2_R agonist treatment. This suggests additional post-transcriptional or post-translational mechanisms may be involved in attenuating the expression of TNF-α protein. Post-transcriptional modification of RNA ultimately determines expression levels by modulating mRNA stability, transport, and translation efficiency. Several key post-transcriptional regulatory elements include RNA-binding proteins, kinases and phosphatases, degradation enzymes, Au-Rich elements (AREs) and microRNA (miRNA). RNA-binding proteins can silence (or facilitate) the translation of TNF mRNA. For example, RNA-binding proteins, T-cell intracellular Ag (TIA-1) and TIA-1-related protein (TIAR), both silence TNF mRNA [44]; kinases and phosphatases change the binding efficiency of RNA-binding proteins and AREs sites. CNS injury alters the mitogen-activated protein kinase (MAPK) pathway enhancing mRNA stability and the efficacy of inflammatory cytokine generation. Interestingly, a CB_2_R agonist was shown to reduce spinal MAPKs (p38 and p-ERK-1/2) through increased expression of mitogen-activated protein kinase phosphatases (MKP-1 and MKP-3) in primary microglial cells [45] and may explain the reduction in TNF-α protein expression in this study. Additionally, post-translational processes that interfere with the release of soluble TNF-α from its membrane-anchored pre-cursor or promote degradation of the protein may be involved.

Circulating leukocytes infiltrate the brain after cortical injury, reaching maximal accumulation 2 to 3 days after injury [19,23,46,47], which coincides with increases in barrier permeability [23]. ICAM-integrin interactions, in particular, are important for leukocyte adhesion and transendothelial migration to the injured brain. ICAM-1 (CD54) is a cell surface ligand that binds the integrins lymphocyte function-associated antigen-1 (LFA-1, CD11a/CD18) and macrophage adhesion molecule (Mac-1, CD 11b/CD 18) [7]. After TBI, increased expression of endothelial ICAM-1 helps to precisely guide the migration of leukocytes to the site of injury [43,48,49]. Increased expression of ICAM-1 is tightly coupled with changes in macrophage/microglia activation after CCI injury [49]. Past and present findings by our laboratory support the notion that stimulation of the CB_2_R attenuates the inflammatory vascular response to injury. ICAM mRNA levels showed a peak at the 1-day time point that was influenced by the CB_2_R deletion but not synthetic blockade. Results indicate a more pronounced effect on the immune-vascular injury response by genetically modifying the endogenous CB receptor system. The process of immune cell infiltration into the damaged parenchyma also enhances the permeability of the BBB. At the site of injury, microglia and accumulating immune cells release pro-inflammatory mediators and free radicals, both of which are known to disrupt the neurovascular unit, compromise the integrity of the BBB, and contribute to excitotoxicity and cell toxicity [8,50,51]. Disruption of the BBB is especially relevant to TBI as it is a proposed secondary injury mechanism creating a cytotoxic environment for neurons [52,53].

Stimulation of the CB_2_R suppresses activation, chemotaxis, and migration of peripheral macrophages, monocytes, and T cells, as well as microglial-like cells [15,16,54-58]. These observations suggest that a number of cell types associated with CNS inflammation express the CB_2_R. Insults to the CNS and pro-inflammatory conditions have been found to result in significant upregulation of CB_2_R mRNA expression [13]. CB_2_R mRNA expression is increased 10-fold by activation of microglia and peripheral macrophages in culture [59]. While there is general agreement that circulating immune cells and microglia express the CB_2_R, a controversy exists over the expression of the CB_2_R on other CNS cell types [14,59-62]. Evidently, this is due to differences in the antibodies used for immunohistochemistry between groups.

Distinctions in the role of infiltrating macrophages and resident microglia in brain injury continues to be a challenge as these cells are both of a monocyte lineage and express many of the same cell-surface markers. Adding to the complexity, is the growing evidence that macrophages and microglia are capable of expressing both pro-inflammatory (M1) and anti-inflammatory (M2) phenotypes [11,12,30]. In addition to pro-inflammatory cytokines and adhesion molecules, iNOS is another marker expressed by a M1 cell phenotype and is useful to study these phenotype differences and injury mechanisms. CB_2_R stimulation significantly reduced iNOS mRNA, while genetic deletion substantially exacerbated iNOS expression. Moreover, qualitative analysis of the remaining cortical tissue surrounding the injury showed iNOS expression to be predominantly from a macrophage/microglia cellular source. In a model of TBI, results suggest that the protection offered by a microglial inhibitor, minocycline, may be through cannabinoid receptors as well [63]. Results suggest that selective modulation of the CB_2_R transforms the inflammatory phenotype of the infiltrating and/or immune cells after injury.

## Conclusion

Modification of the inflammatory vascular responses to TBI through CB_2_R stimulation, as well as receptor blockade and deletion demonstrate the importance of this receptor in recovery from TBI. The CB_2_R is an endogenous regulator of the inflammatory response to TBI, working at the interface between the brain and microvasculature. Macrophage/microglial modulation using CB_2_R agonists has a significant contribution to its protective capabilities [18,24,25]. Interventions that limit prolonged microglial activation and neuroinflammation as occurs after TBI, or facilitate a reparative microglial phenotype are proposed to counteract the development of cognitive and affective disorders [30]. Our results further support that CB_2_R-dependent pathways regulate the bridge between immune and vascular function following TBI. The development of pharmacological agents to treat TBI may rest in furthering our understanding of the complex immune, vascular, and nervous system interactions that are induced at the time of injury.

## Abbreviations

TBI: traumatic brain injury
CCI: controlled cortical impact
CB_2_R: cannabinoid receptor type-2
TNF-α: tumor necrosis factor-alpha
ICAM-1: intracellular adhesion molecule-1
iNOS: inducible nitric oxide synthase
BBB: blood-brain-barrier
KO: knockout
ELISA: enzyme-linked immunosorbent assay
